# Keratoconus Management With Customized Photorefractive Keratectomy by Artificial Intelligence Ray-Tracing Optimization Combined With Higher Fluence Corneal Crosslinking: The Ray-Tracing Athens Protocol

**DOI:** 10.1097/ICO.0000000000002739

**Published:** 2021-05-26

**Authors:** Anastasios John Kanellopoulos

**Affiliations:** * Department of Ophthalmology, the Laservision.gr Clinical and Research Institute, Athens, Greece; and; † Department of Ophthalmology, New York University, School of Medicine, New York City, NY.

**Keywords:** ray-tracing excimer customization combined with corneal crosslinking (CXL), higher fluence crosslinking, accelerated crosslinking, keratoconus, Athens protocol, topography-guided excimer ablation for keratoconus

## Abstract

**Purpose:**

The aim of this study was to report novel ray-tracing customization of surface excimer laser ablation combined with higher fluence corneal crosslinking (CXL) in the stabilization and normalization of ectasia and visual rehabilitation of progressive keratoconus.

**Methods:**

A 28-year-old man with bilateral progressive keratoconus was treated with Athens protocol: CXL combined with photorefractive surface ablation customized by a novel artificial intelligence platform calculating lower- and higher-order aberrations based on wavefront, Scheimpflug tomography, and interferometry axial length data from a single diagnostic device. Visual acuity, refractive error, keratometry, optical coherence tomography and Scheimpflug tomography, and endothelial cell density were evaluated over 12 months.

**Results:**

Keratoconus stabilized in both eyes. Uncorrected distance visual acuity changed from 20/80 to 20/20 in the OD and from 20/40 to 20/25 in the OS at 12 months. Keratometry changes were as follows: from 40.7 and 42.7 at 165.1 degrees to 41.4 and 43.1 at 169.3 degrees in the OD and from 40.9 and 42.6 at 15.9 degrees to 44.1 and 44.7 at 9.8 degrees in the OS. Corneal surface normalization was as follows: index of height decentration from 0.115 to 0.099 and index of surface variance from 77 to 67 in the OD and index of height decentration from 0.066 to 0.014 and index of surface variance from 49 to 31 in the OS.

**Conclusions:**

We introduced in this study the management of progressive keratoconus with CXL combined with novel excimer laser customization using several independent up-to-now diagnostics calculated by software, evaluating bidirectional theoretical ray tracing. It bears the potential advantage of addressing more accurately normalization of the distorted human eye optics associated with corneal ectasia, compared with using anterior corneal surface data or wavefront data alone.

Keratoconus and corneal ectasia have been managed with a paradigm shift over the past 2 decades with the advent of corneal crosslinking (CXL).^1^ We introduced and have since reported extensively on the use of higher fluence ultraviolet light for accelerated CXL in keratoconus.^2,3^ Among the many subsequent treatments and technique innovations introduced, CXL almost invariably results in 1- to 2-diopter (D) central anterior corneal flattening, described as disease regression.

Almost alongside, we introduced^4^ and have reported extensively on combining a partial in-refraction, topography-guided surface ablation technique to combine corneal normalization and CXL as a means to not only stabilize ectasia but also significantly reshape the irregular corneal surface, facilitate riboflavin solution pharmacokinetics within the corneal stroma, enhance the CXL photochemical reaction, and, as an end point, improve visual function. We termed the combination technique as the Athens protocol.^5,6^

We recently reported on long-term 10-year data of cases we had treated with the Athens protocol,^7^ in addition to long-term data in using the Athens protocol in pediatric cases.^8^ Other investigators have also reported on combining a customized partial refraction surface ablation and CXL in the management of keratoconus and ectasia.^9^

In addition, wavefront-guided photorefractive keratectomy (PRK) normalization has been also reported as a combination treatment along with CXL for keratoconus.^10–12^ Ray tracing has been described as a means of customization for routine myopic excimer treatments, using bimodal calculation of light incidence through the human eye, studied with the aid of wavefront, anterior segment tomography, and interferometry axial length data. We present in this study a modification of the Athens protocol for progressive keratoconus, using artificial intelligence–generated ray-tracing calculation of the excimer laser–customized ablation aiming to normalize the distorted optics by the ectasia.

## METHODS

This study received approval from the ethics committee of our institution (The Laservision Ambulatory Care Center Ethics Committee, session number 114, September 23, 2019, pages 96–98) and adhered to the tenets of the Declaration of Helsinki. Written informed consent was obtained at the first study visit. All equipment, techniques, and materials at the time of treatment had been already approved for clinical use in myopic laser vision correction within the EU (CE mark). Its use in irregular corneas was off-label. The combination technique of using ray-tracing customization combined with CXL was novel and not the technologies used.

**FIGURE 1.**
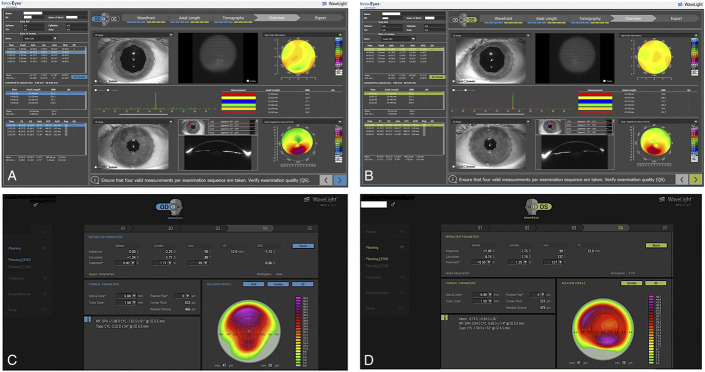
The Sitemap diagnostic device summary display of measurements captured for the OD (A) and the OS (B): wavefront data in the top row of images, interferometry axial length data in the middle, and tomography data in the bottom. The InnovEyes treatment interface summary display for the OD (C) and the OS (D): In both (C, D) images, first line shows the subjective refraction, middle line the calculated refraction by the software, and bottom line the adjusted treatment used for the customized surface ablation. We manually chose to treat less hyperopia than the manifest refraction and that calculated by the device because we anticipated from long experience with the Athens protocol technique that the 40-μm PTK would induce a myopic shift.

The Sitemap device is in essence an adapted Pentacam AXL Wave device (Oculus, Germany), which, as a single diagnostic device, provides the captured data of multiple evaluations: wavefront, Scheimpflug tomography, pupillometry, and iris recognition data and interferometry axial length measurements. Figures 1A, B illustrate the summary data acquired for both eyes reported in this study with the Sitemap device. The InnovEyes artificial intelligence software is a novel customization option in the existing platform of ablation options with the EX500 excimer laser (Alcon, Wavelight, Erlagen, Germany) that has received CE mark (Conformitee European) for clinical use within the countries of the European Union for the treatment of myopic astigmatism. It processes these data once they are exported and transferred from the Sitemap device through a proprietary stand-alone Ethernet network to the EX500 excimer treatment design and delivery laptop computer.

The ray-tracing technique by the InnovEyes platform is described in the following steps:

Step 1: The axial length and keratometry measurements provided by the interferometry device within the Sitemap unit give precise positions of all anatomical refractive surfaces along the line of sight so that a theoretical virtual model eye is constructed with them to proceed to the next steps. This is a unique feature in customized treatment planning because all other previously released customized platforms use the Gullstrand eye model.

Step 2: The corneal tomography measurements are used to calculate the propagation of light rays (2000 in number) through the anterior corneal surface, through the corneal stroma, out the posterior corneal surface, through the anterior chamber, and onto the anterior lens surface.

Step 3: The wavefront data are used to calculate each of the aforementioned light ray travel in a retrograde fashion: from the retina, through the vitreous cavity, onto the posterior lens surface, through the crystalline lens, and onto the anterior surface of the lens. A total of 2000 rays are actually traced and calculated in these 2 steps for the algorithm determination. The system requirement for this is a mesopic/scotopic capture that achieves at least 6 mm of pupillary dilation by the patient. Technically, this is the reason that during the capture, the wavefront is performed first in both eyes to avoid fatigue and miosis in patients, followed by tomography and interferometry in 1 eye and then in the other.

Step 4: In the final fourth step, tilt is calculated by the Scheimpflug tomography images and the incidence of rays projected onto the anterior surface of the cornea. The internal tilt between the actual anterior corneal surface orientation toward the ray-tracing orientation used in steps 2 and 3 is additionally taken into consideration and corrected by the InnovEyes artificial intelligence software. The software attempts to “line-up” the corneal surface to the whole eye orientation used in the ray-tracing steps 2 and 3, thus adjusting the corneal surface by applying a prism-like ablation to perform the higher-order aberrations correction measured on the optimally conformed anterior corneal surface.

The patient presented in this study was evaluated 7 months earlier with bilateral mild keratoconus and advised to stop eye rubbing and present for reevaluation in 6 months. Seven months later, he presented with symptoms of worsened visual function and change in keratometric values from 41.9 and 41.1 at 85 degrees to 42.7 and 40.7 at 77.1 degrees in the OD and from 42.0 and 41.5 at 120 degrees to 42.6 and 40.9 at 115.9 degrees in the OS. Minimal corneal thickness reduced from 475 to 465 μm and from 498 to 482 μm in the OD and OS, respectively; the aforementioned measurements were performed with Scheimpflug-based tomography.

Each eye of the 28-year-old man with bilateral progressive keratoconus was treated initially with the ray tracing–guided PRK (InnovEyes) planned ablation as follows: The actual customized PRK treatment was plano −1.71 at 89 degrees applied over a 6-mm optical zone and 1.5-mm transition zone using the WaveLight EX500 excimer laser (Alcon, Fort Worth, TX). The corresponding manifest clinical refraction was +1.25 to 2.25 at 95 degrees with uncorrected distance visual acuity (UDVA) of 20/80 and CDVA of 20/30. The adjustment of the refraction used was based on our experience of the corneal reshaping usually inducing a myopic shift of 1 to 2 D, possibly from steepening the superior to the center cornea and/or the myopic shift associated with phototherapeutic keratectomy (PTK) aimed to remove the epithelium, as noted further. After the customized InnovEyes ablation, a 40-μm depth and 8-mm diameter PTK was ablated to account for epithelium removal. The 40-μm PTK depth is based on the standard Athens protocol and has been derived from clinical observation through hyperfluorescence during epithelial removal when used first on the naive corneal surface, which showed that it suffices for complete epithelial removal when this diameter and ablation depth are used. It was clear during this stage of ablation that some peripheral stromal tissue was removed, and we certainly expected that this PTK may even remove some central stromal tissue beyond the expected volume of epithelium targeted to be removed. At the end of PTK, a 20-second soaking with a wet Weck-Cel sponge soaked in 0.02% Mitomycin-C on the exposed corneal stroma was performed. The next step was a 5-minute soaking with 0.1% riboflavin solution administered on the corneal surface 1 drop every 30 seconds (VibeX Rapid, Avedro, Waltham, MA). The corneal thickness was measured then to be more than 400 μm by the optical pachymetry measurement provided by the EX500 excimer laser. Then, the KXL I device by Avedro (Glaucos, San Clemente, CA) was engaged over the cornea, and the CXL treatment took place, while riboflavin drops were administered every 1 minute for the duration. The UVA radiation with fluence of 10 mW/cm^2^ delivered at a 7-mm diameter centered on the pupillary aperture was administered for 10 minutes for a total of 6 J of UVA energy.

The OS was treated with the same technique, and the customized ray-tracing treatment was plano −1.25 at 137 degrees. The clinical manifest refraction in the OS was +1.00 to 1.75 at 90 degrees with UDVA of 20/40 and CDVA of 20/25 but with significant ghosting of the optotype numbers described subjectively by the patient more in the right eye than that in the left. Figure 1 illustrates the actual InnovEyes ray-tracing PRK for each eye. The maximum ablation depth was 47 μm in both eyes and an estimated 36 and 42 μm over the thinnest point of cornea of the OD and OS, respectively, as illustrated in Figure 1.

At the completion of both CXL treatments, a bandage contact lens (+0.50 D, base curve: 9.2 and 14 mm diameter; AcuVue, Johnson & Johnson) was placed on the cornea along with 1 drop of moxifloxacin (Vigamox, Alcon) and 1 drop of a combination of 1% dexamethasone and 0.3% chloramphenicol (Dispesadron-C, Alcon). The postoperative regimen was continued 4 times a day for 1 week, and then the dexamethasone/chloramphenicol combination drop for 1 month, which was replaced by Lotemax drops twice a day for the second postoperative month.

Follow-up visits were at 1 to 5 days, 1, 2, 3, and 6 months, and 1 year. The cornea reepithelialized by day 4, and the bandage contact lens was removed from both eyes. Visual acuity, refractive error, corneal clarity, keratometry, topography, and pachymetry with a multitude of modalities and endothelial cell density were evaluated over 12 months.

## RESULTS

Keratoconus stabilized in both eyes. The keratoconus stage by Amsler-Krumeich criteria remained at 2 in the OD and improved from stage 1–2 to stage 1 in the OS. UDVA changed from preoperative 20/80 to 20/20 in the OD and from 20/40 to 20/25 in the OS at 12 months. Keratometry changes were as follows: from 40.7 and 42.7 at 165.1 degrees to 41.4 and 43.1 at 169.3 degrees in the OD and from 40.9 and 42.6 at 15.9 degrees to 44.1 and 44.7 at 9.8 degrees in the OS. Corneal surface normalization became more evident in the OS with index of height decentration [index of height decentration (IHD)—an anterior corneal curvature index of asymmetry to the measured corneal vertex] improvement from 0.066 to 0.014 and index of surface variance from 49 to 31 The corresponding changes in the OD were IHD from 0.115 to 0.099 and index of surface variance from 77 to 67.

**FIGURE 2.**
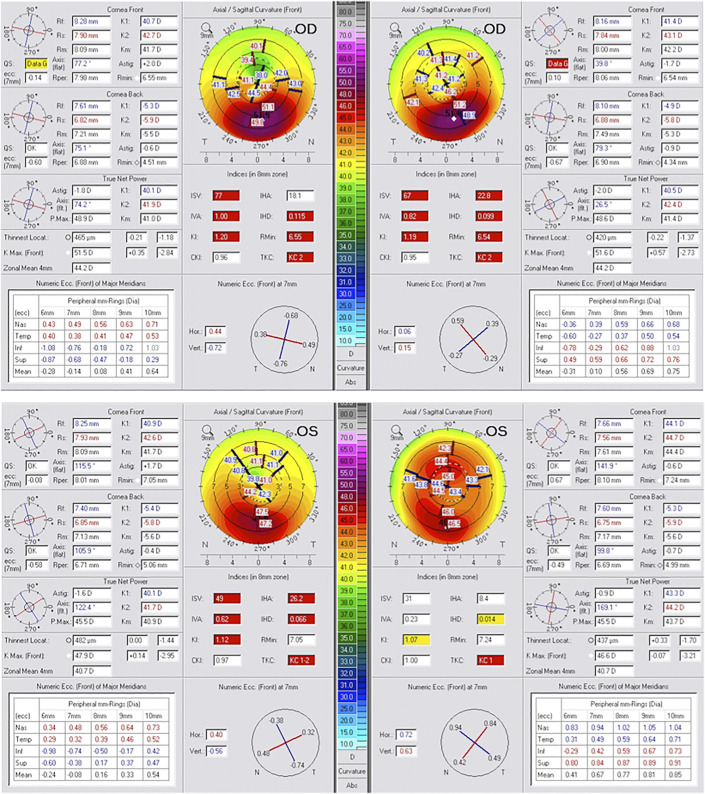
Scheimpflug imaging data showing comparison of the axial/sagittal front curvature tomography keratometric data including K1, K2, Km, Astig, Rmin, and axis; thinnest local, Kmax, and zonal mean of 4 mm; tables with eccentricity data; and the topometric asymmetry indices including index of surface variance, index of height asymmetry, index of vertical asymmetry, IHD, K1, and Rmin preoperatively (left) and at 6 months postoperatively (right) for both eyes (OD, top; OS, bottom).

The aforementioned and all other Scheimpflug tomography–derived anterior curvature asymmetry indices changes from preoperatively to 12 months postoperatively are shown in Figure 2. The comparison maps demonstrated not only cone flattening in both eyes, being more prominent in the OS, but also for the normalization process the significant simultaneous steepening of the central corneal area just superior to the cone, resulting in overall normalization—the key feature, in our opinion, in the clinical benefit of using the Athens protocol CXL technique, and its ability to, beyond ectasia stabilization, also improve drastically central corneal symmetry and, as a result, visual function regarding CDVA and quality of mesopic and scotopic vision.

**FIGURE 3.**
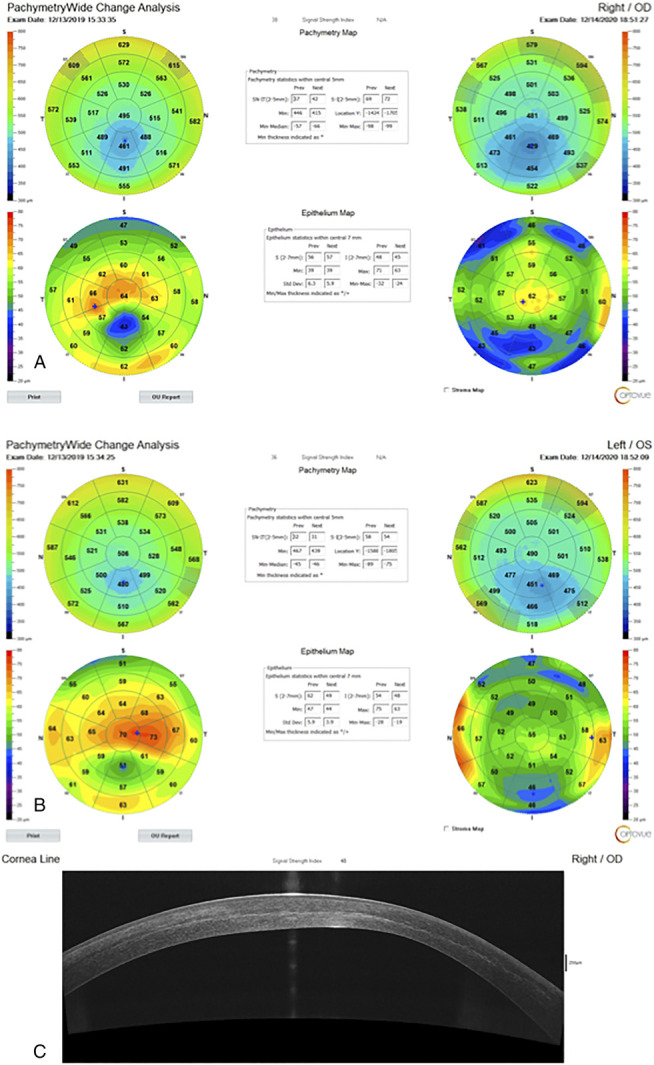
Anterior segment OCT-derived total corneal thickness mapping and epithelial mapping showing comparison of preoperative with those of the 12-month postoperative for the OD (A) and the OS (B). C, In a section of the OD at 12 months, the CXL line at more than 50% depth within the stroma, an imaging sign of the depth and width of the CXL effect.

In Figure 3, an anterior segment optical coherence tomography (OCT)-derived total corneal thickness mapping and epithelial mapping comparison of preoperative with those of the 12-month postoperative demonstrates the overall thinning of the cornea to be from 446 to 415 μm for the thinnest point in the OD (top image A) and from 467 to 439 μm in the OS, respectively (middle image B), whereas the epithelial maps have dramatically normalized in both eyes, possibly masking the extent of stromal normalization that was accomplished. We used clinically the “thickening” to normal and above-normal epithelium over the thinnest part in the total thickness corneal map postoperatively, following the Athens protocol CXL, because of clinical evidence of corneal stability and noteworthy changes from the typical epithelial remodeling in ectasia, which were also evident in both eyes of this patient preoperatively: much thinner over the cone and much thicker around it. Figure 3C demonstrates in an OCT section line the CXL line at more than 50% depth within the stroma, an imaging sign of the depth and width of the CXL effect.

In the section image of the treated cornea at month 12 (Optovue RT-Vue device, CA), there is a clear demonstration of a very deep CXL line, supporting evidence for the effective depth and width of the CXL effect accomplished.^13^ The endothelial cell counts were essentially unchanged in both eyes, with the preoperative values being 2720 and 2850 cells/mm^3^ and postoperative being 2750 and 2835 cells/mm^3^ in the OD and OS, respectively.

## DISCUSSION

Significant refractive benefit has been documented with traditional CXL, when applying the Dresden protocol (3 mW/cm^2^ for 30 min), and with higher fluence or otherwise described as accelerated CXL, introduced by our investigative team in Athens, Greece, and subsequently reported on both in vivo and ex vivo studies. The reshaping of the irregular ectatic cornea, by corneal flattening at the apex and steepening at the irregularly flattened central and superior, usually paracentral, corneal area, most probably constitutes a refractive effect resulting from differential crosslinking–induced stiffening effects within the stromal collagen of the same cornea, allowing the cornea to return to previous more regular shape, which has been described in the past by a number of clinicians as disease regression.

The combination of CXL in eyes treated with the Athens protocol aims to enhance the corneal normalization, achieved by the refractive effect of the partial in-refraction topography-guided PRK. This is evident by the much larger corneal keratometric changes achieved when one considers the respective change expected when applying the Munnerlyn formula.

When compared with our previously reported data on applying the Athens protocol in ectasia and keratoconus,^2,4–8^ the data in this case suggest also a significant refractive effect, with high levels of corneal normalization as noted by the IHD change, especially in the OS, and the functional improvement of the UDVA and CDVA achieved in both eyes, along with effective CXL documentation such as the deep CXL demarcation line on anterior segment OCT imaging. Of course, these findings underline feasibility and safety in just 2 cases and cannot form conclusive evidence, enough for comparison with studies that include many more cases and longer follow-up.^7^

It is of interest that the designed ablation with slightly myopic spherocylindrical equivalent, combined with the documented flattening effect of CXL, results in postoperative refraction that is slightly less hyperopic than preoperative. The explanation in our experience lays in converting the initially multifocal central cornea (steep over the cone and flatter just superior to it) to a more uniform in central keratometric power: the cone flattens and the flatter superior area to the cone steepens. This is the most probable reason for, despite the flattening effect in the cone area (in some of our reported cases, even more than 10 D in power), the postoperative refractive error to experience a myopic shift because the central corneal lens becomes multifocal and possibly because there is a concomitant centroid shift in the patient's line of sight.

Ray tracing has been described for more than 10 years as a means of refractive outcome optimization using manual calculations from the aforementioned principles of wavefront, corneal topography, and interferometry axial length measurements, performed at the time and until recently with mainly manual calculations by an expert team and specific protocols.^12^ It has never been reported, to our knowledge, as a combination treatment for keratoconus eyes along with CXL. We described in this study, to our knowledge, the first clinical data with ray tracing processed by artificial intelligence and with measurements from a single novel multidiagnostic device, used for the first time in irregular eyes such as corneal ectasia in combination with CXL.

The ability to include total eye refractive and aberration data in the normalizing therapeutic PRK may prove to overcome several unknowns when compared with our previous experience of 15 years in managing these irregular eyes with anterior corneal curvature data alone. When using the Placido-derived topographic data to normalize ectatic corneas, the surgeon is called to forecast expected refractive changes that will take place, which in our experience have been mainly a myopic shift.

In addition, the ray tracing–customized ablation will in theory consume less stromal tissue compared with the pure anterior surface topography–guided ablation because the anterior surface component that compensates for the posterior corneal irregularity will not be ablated.     The posterior corneal surface changes associated with ectasia may contribute as a major source of deteriorated corneal optics, and the ablation constructed by reverse ray tracing may better address the limitation of changing just the anterior surface irregularity.

Potential limitations of this novel technology described in this study may include the ability to potentially image some of these highly irregular eyes through more ectatic and distorted corneas and the amount of tissue suggested to be treated in consideration along with the minimum available stromal thickness present at each case. Because we have treated (unpublished data presented at the 2020 annual ASCRS, ESCRS, and AAO meetings) several cases with moderate keratoconus that were able to be imaged and calculated with this technology, we theorize that ray tracing–automated optimization may be applicable to a wide range of cases with mild to even moderate keratoconus.

Another limitation is the potential lenticular changes that may coexist, especially in older patients, which may significantly bias the normalization calculations by this ray-tracing technique to include crystalline lens change data. Clinicians need to recognize and use caution in such cases. These issues may be addressed by an only-corneal reverse ray tracing–based ablation, a technique that can become useful and a platform that should be, in our opinion, an expectation from the industry.

Additional studies of more cases and longer follow-up may further validate the data presented in this preliminary feasibility study. We in this study introduced a novel technique based on the combination of higher fluence CXL along with customized PRK (Athens protocol) using ray tracing from artificial intelligence that combined data from wavefront, Scheimpflug tomography, and interferometry axial length measurements for safe and effective management of progressive keratoconus in young adult patients.
